# Formation processes for large ejecta and interactions with melt pool formation in powder bed fusion additive manufacturing

**DOI:** 10.1038/s41598-019-41415-7

**Published:** 2019-03-25

**Authors:** Abdalla R. Nassar, Molly A. Gundermann, Edward W. Reutzel, Paul Guerrier, Michael H. Krane, Matthew J. Weldon

**Affiliations:** 1 The Pennsylvania State University Applied Research Laboratory, P.O. Box 30, State College, Pennsylvania, PA 16804-0030 USA; 2 Moog Inc. 500 Jamison Rd., Plt. 20, East Aurora, NY 14052 USA

## Abstract

Ejecta with a size much larger than the mean particle size of feedstock powder have been observed in powder bed fusion additive manufacturing, both during post-process sieving and embedded within built components. However, their origin has not been adequately explained. Here, we test a hypothesis on the origin of large (much larger than the mass-median-diameter of feedstock powder) ejecta—that, in part, they result from stochastic, inelastic collisions of ejecta and coalescence of partially-sintered agglomerates. The hypothesis is tested using direct observation of ejecta behavior, via high-speed imaging, to identify interactions between ejecta and consequences on melt pool formation. We show that stochastic collisions occur both between particles which are nearly-simultaneously expelled from the laser interaction zone and between particles ejected from distant locations. Ejecta are also shown to perturb melt pool geometry, which is argued to be a potential cause of lack-of-fusion flaws.

## Introduction

Laser powder bed fusion additive manufacturing (PBFAM), a subcategory of metal 3D printing technology, is rapidly emerging as an important industrial manufacturing technology for aerospace, medical, and defense applications^1,2^. The process, which relies on sequential melting of powder layers on the order of tens of microns in thickness, is useful for the production of complex, previously un-manufacturable, geometries. However, significant challenges remain in understanding the complex material transfer and heat transfer mechanisms which take pace during the many melting and re-melting cycles during processing. In particular, the mechanism leading to the formation of ejecta (e.g. spatter), commonly observed during processing, and their influence on build quality remains in dispute.

To date, little is known regarding the mechanisms by which spattered particles form or how they influence flaw formation processes in powder bed fusion additive manufacturing. Models of the PBFAM process have proven useful in better understanding melt pool dynamics and in showing that melt ejection and powder denudation play important roles in the process^3,4^. More recently, high-speed imaging has been used to argue that ejecta primarily forms, not as a result of melt ejection, but due to evaporation-driven entrainment of powder^5^. Though, according to Ly *et al*.^5^, when melt ejection does occur in PBFAM, it tends to produce ejecta on the order of 25–100 μm in size. This is because the kinetic energy of ejected melt must exceed the capillary pressure of the melt (surface tension divided by the melt’s radius of curvature). Hence, larger melt droplets are more likely to be ejected; powder enhances this effect by hindering forward motion of the melt. Nevertheless, entrained particles—of a similar size distribution as the feedstock powder particles—are argued to form approximately 85% of spatter. Of this portion, about 60% are described as “hot”^5^.

High-speed X-ray imaging of the PBFAM process^6^ reinforces, and adds to, the findings of Ly *et al*.^5^. Again, evaporation-driven entrainment of particles, due to flows of metal vapor and ambient gas, is identified as the primary mechanism for spatter formation. The proportion of hot particles also appears to depend on environmental pressure^6^. Interestingly, both works^5,6^ maintain that larger spatter particles are likely due to melt ejection, while entrained particles have similar size distributions to the feedstock powder. Though it should be noted that Ly *et al*.^5^ do show the formation of large particles through the collision and merging of neighboring, hot droplets expelled from the melt pool. More recently, Bidare, Bitharas, *et al*.^7,8^ used high-speed imaging and modeling to demonstrate the complex nature of plume-powder-melt interactions and argue that sintering and melting of denuded particles contributes to the formation of large ejecta. Additionally, their work^7^ shows that coalescence of partially-sintered agglomerates can occur due to entrainment of agglomerates into the vapor plume and subsequent melting. Another mechanism, suggested by Körner, Bauereiss *et al*.^9,10^ based on simulations of electron-beam PBF, involves melting and coalesce of powder, at the edge of electron-beam-melted tracks, by interaction with superheated melt droplets.

It has been speculated that ejecta atop the powder bed may contribute to flaws in one of three ways: (1) spatter particles dragged during powder recoating may perturb the powder bed causing height variations^11^; (2) large spatter particles may remain un-melted and become incorporated into the component^12,13^; or, (3) large ejecta may shadow the beam and thus contribute to lack-of-fusion defects^10^. Among these potential mechanisms, (2), and (3) are more likely since perturbations in powder height are not an uncommon occurrence in PBFAM, typically resulting from interactions of the recoater with elevated or thermally-deformed parts on the build plate^14,15^. It is also not generally accepted that such powder perturbations lead to defects. In contrast, it has been shown^12^ that flaws containing unmelted powder, and an associated reduction in tensile strength, result from processing with powder contaminated with a large number of spattered particulates that are roughly three times the diameter of virgin powder. It is therefore critical for the formation of defect-free PBFAM components that the mechanism for the formation of large ejecta be understood and, if possible, mitigated.

Our results indicate that melt ejection is not the sole mechanism for the formation of large ejecta particles. Rather, we show that large ejecta (much larger than the mass-median-diameter of feedstock powder) form as a result of stochastic, inelastic collisions of ejecta and coalescence of partially-sintered agglomerates. We present evidence that stochastic collisions occur both between neighboring particles, defined as those which are nearly-simultaneously expelled from the laser interaction zone, and between distant particles, defined as those ejected from distant locations. These results reinforce some of the findings of Ly *et al*.^5^, who reported collision and merging of nearly-simultaneously expelled particles, and the findings of Bidare *et al*.^7^, who reported coalescence of partially-sintered agglomerates via entrainment into the vapor plume. Also, for the first time, large ejecta are also shown to interfere with the intended melt geometry, which is argued to be a potential cause of lack-of-fusion flaws.

## Results and Discussion

The primary results of this work are elucidations of mechanisms for the formation of large ejecta and evidence that such ejecta affect melt pool geometry. Here, two mechanisms for the formation of large, spheroidal ejecta are investigated: stochastic, inelastic collision of ejecta and coalescence of partially-sintered agglomerates. Evidence of interference of large ejecta with melt pool formation is also reported and argued to contribute to the formation of lack-of-fusion flaws.

### Stochastic agglomeration of ejecta

The formation of large, spheroidal ejecta was observed to occur via multi-body collisions. Collisions were observed to occur between particles ejected from distant locations as well as between neighboring, near-simultaneously-expelled ejecta.

**Figure 1:**
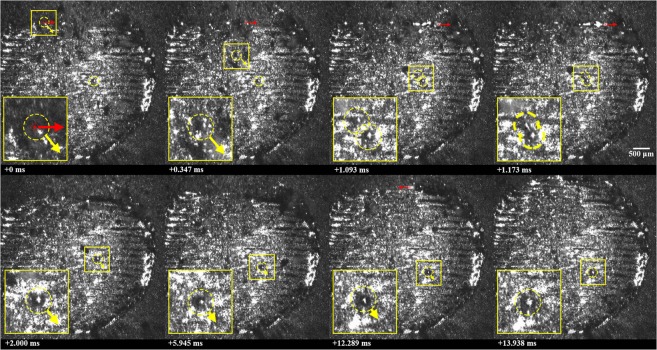
Collision between particles ejected from distant locations, where a molten ejectum is expelled from the laser interaction zone and inelastically collides with a previously-expelled ejectum sitting on the surface of the part. Ejecta are highlighted using a yellow, dashed circle; the location of the laser beam is highlighted using a red ⊕ symbol; the motion of the laser and particles are shown using arrows, a 2.5x magnified image is shown to the bottom left of each frame. Note that a time stamp is indicated on each frame, relative to the first frame, and laser is off during frames 5, 6 and 8.

**Figure 2:**
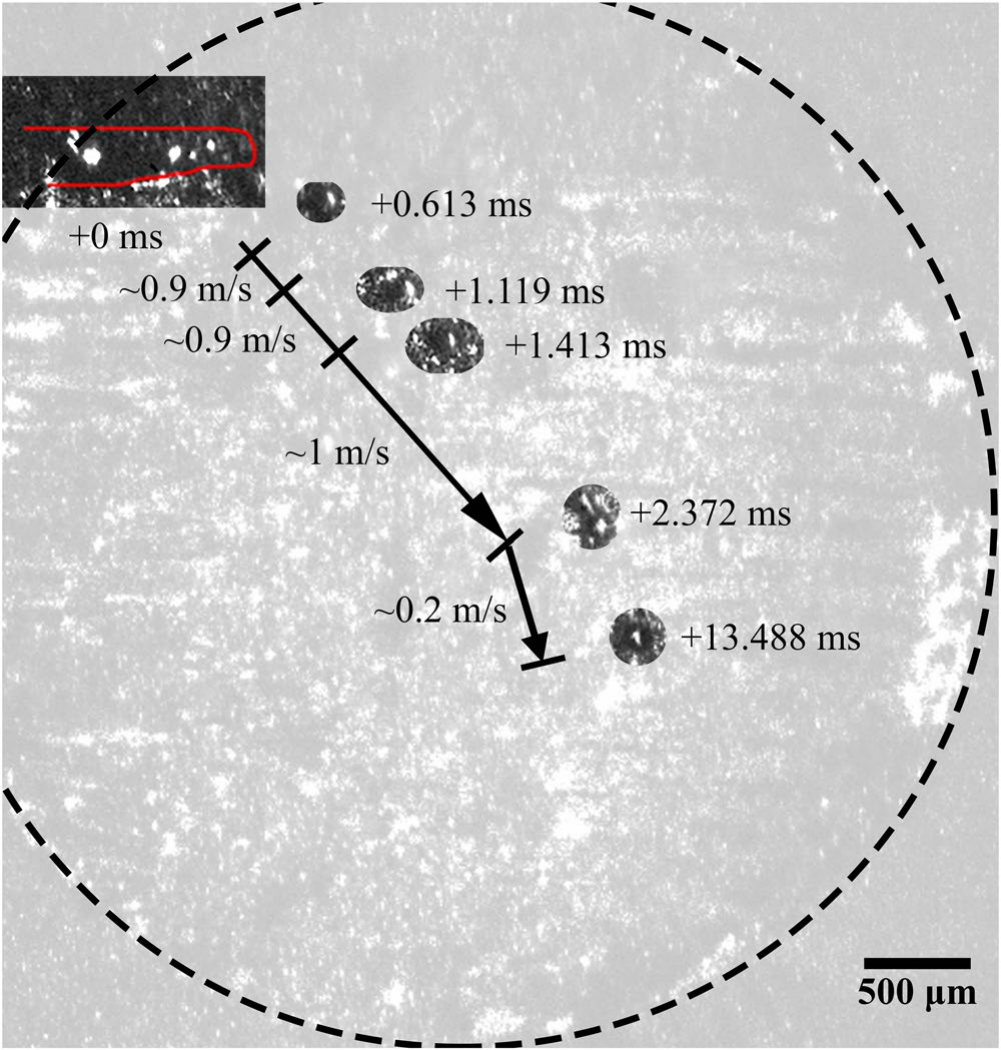
Composite image of expulsion of partially-molten ejectum from laser interaction zone and collision with previously-expelled ejectum. Relative time stamps along with the direction and speed of the ejecta are presented along with the outline of part (dashed line) and the melt pool geometry (solid, red line).

A collision between particles ejected from distant locations is shown in Fig. 1 (See Supplementary Video S1), where a molten ejectum, approximately 88 μm in diameter, is expelled from the laser interaction zone and inelastically collides with a previously-expelled, and now solidified, ejectum sitting on the surface of the part. It should be noted that the powder bed was perturbed to the right of the cylinder, as describe in the methods section, prior to processing this layer. Expulsion takes place from the front, right side of the laser-interaction zone at an angle of approximately 45 degrees relative to the laser translation direction. The expelled particle moves at a near constant speed of 1 m/s until colliding with a stationary, already-solidified particle, measuring approximately 100 μm in diameter and located approximately 2 mm away from the laser-interaction zone. The collision is completely inelastic and results in the formation of a larger agglomerate which comes to rest at a location approximately 0.5 mm away from the location of the collision. A composite image of the expulsion and trajectory of the ejecta towards the previously-spattered particle is provided in Fig. 2.

Immediately after collision, the spheroidal agglomerate moves at a speed of approximately 0.2 m/s. Given the comparable sizes of the particles and the speed of the expelled particle, this is a significantly slower speed than can be expected for a completely inelastic collision of two solid particles. Additionally, the trajectory of the agglomerate is slightly altered after collision. Both observations perhaps indicate that the previously-expelled ejectum may have been partially-sintered to the underlying substrate. Argon shielding gas, directed from the bottom to the top of each image frame at an approximate speed of 1.5 m/s, may also have an influence on the trajectory and speed of the particle^16–18^ and agglomerate, though any effects appear to be minor in this case.

**Figure 3:**
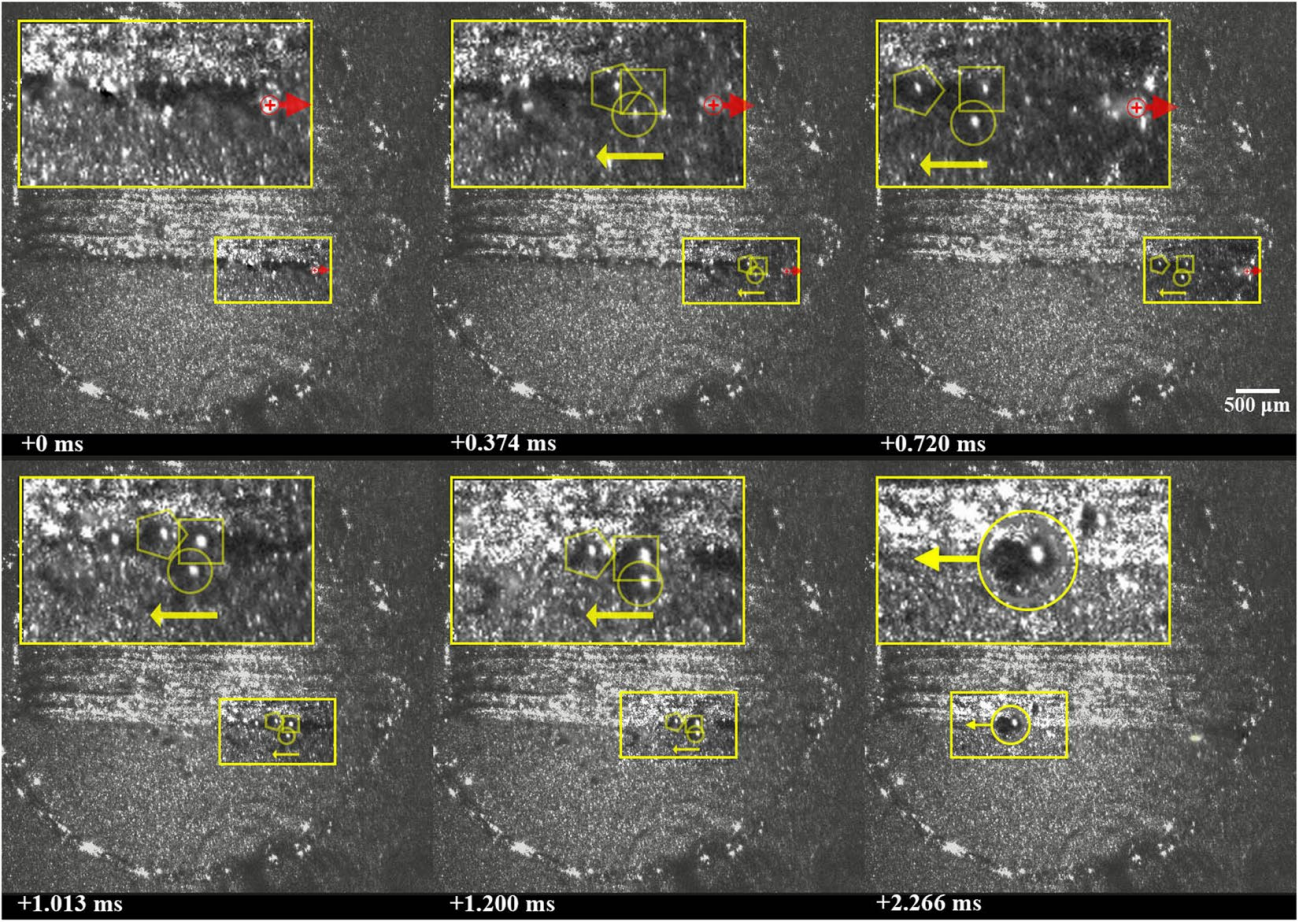
Formation of a large agglomerate via three-body interaction of molten ejecta. Each molten ejectum is highlighted using a yellow pentagon, square, and circle; the location of the laser beam is highlighted using a red ⊕ symbol; the motion of the laser and particles are shown using arrows; the final large agglomerated particle is highlighted with a yellow circle. A 2.5x magnified image is shown to the top left of each frame. Note that laser is off in frames 4–6.

Collision between neighboring, near-simultaneously-expelled ejecta is a second mechanisms identified for the formation of large, spheroidal agglomerates. An example of this phenomena is provided in Fig. 3 (See Supplementary Video S2), wherein three spherical ejecta are expelled backwards, relative to the direction of laser scanning. Within a period of approximately 2.27 ms, the three particles, each ranging from 102 to 143 μm in diameter, collide and coalesce to form a single, spheroidal agglomerate measuring approximately 190 μm in diameter. A faint shadow is observed below each ejectum, indicating that each was expelled upwards relative to the surface of the cylinder. Following collision, the ejecta form a single, spheroidal agglomerate; this indicates that they are at least partially-molten before and during collision.

**Figure 4:**
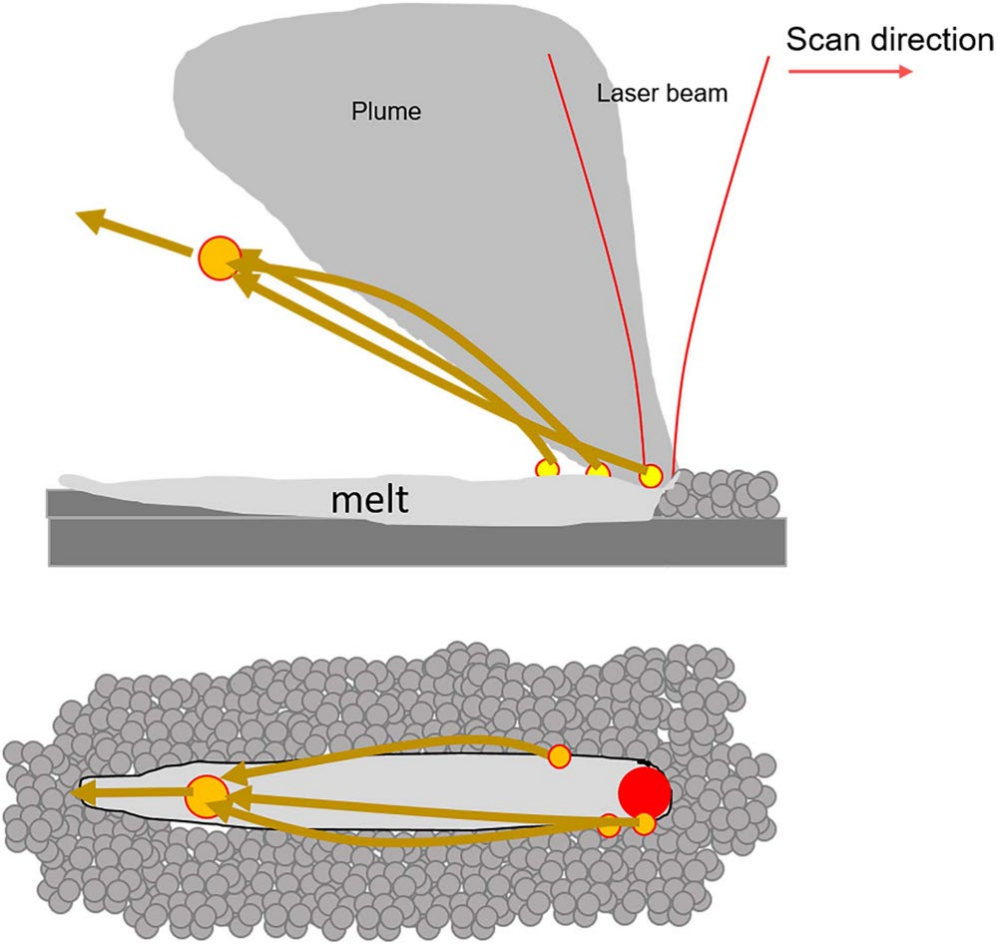
Illustration of expulsion of multiple neighboring particles and their collision to form an agglomerate.

Careful analysis of the high speed video data appears to show that ejecta emerge from along the sides of the laser-interaction zone and are accelerated backwards, relative to the direction of laser scanning. Based on their smoothness and dark appearance, which indicate significant reflection of the illumination laser, the ejecta are at least partially molten when they emerge from the laser-interaction zone and after collision. It is unclear if the ejecta are a product of powder entrainment into the vapor or melt ejection. In either case, the backwards acceleration and seemingly curved path of the particles appears to be at least partially due to the influence of the vapor plume, as illustrated in Fig. 4. Argon shielding gas, directed from the bottom to the top of each image frame at an approximate speed of 1.5 m/s, may also have an influence on the trajectory of the particles. The effect of shielding and the laser scanning direction relative to it have been shown to significantly affect the landing location of ejecta^16–18^. It is noteworthy that in this case, the velocity of the spheroidal agglomerate is perpendicular to the shielding gas, and appears to be only slightly affected by the shielding gas within the observable field of view. Given the upward trajectory of the agglomerate, the shielding gas may affect the ultimate landing location of the agglomerate^16,17^.

It ought to be noted that the formation of a large agglomerate through the collision of neighboring, near-simultaneously-expelled ejecta has previously been shown in supplementary high-speed images accompanying the works of Bidare, Bitharas, *et al*.^8^ Though, this was not explicitly called out by the authors. Ly *et al*.^5^ have also documented the formation of large particles through the collision and merging of neighboring, hot droplets expelled from the melt pool, though over a much shorter distance than presented here.

### Coalescence of partially-sintered agglomerates

**Figure 5:**
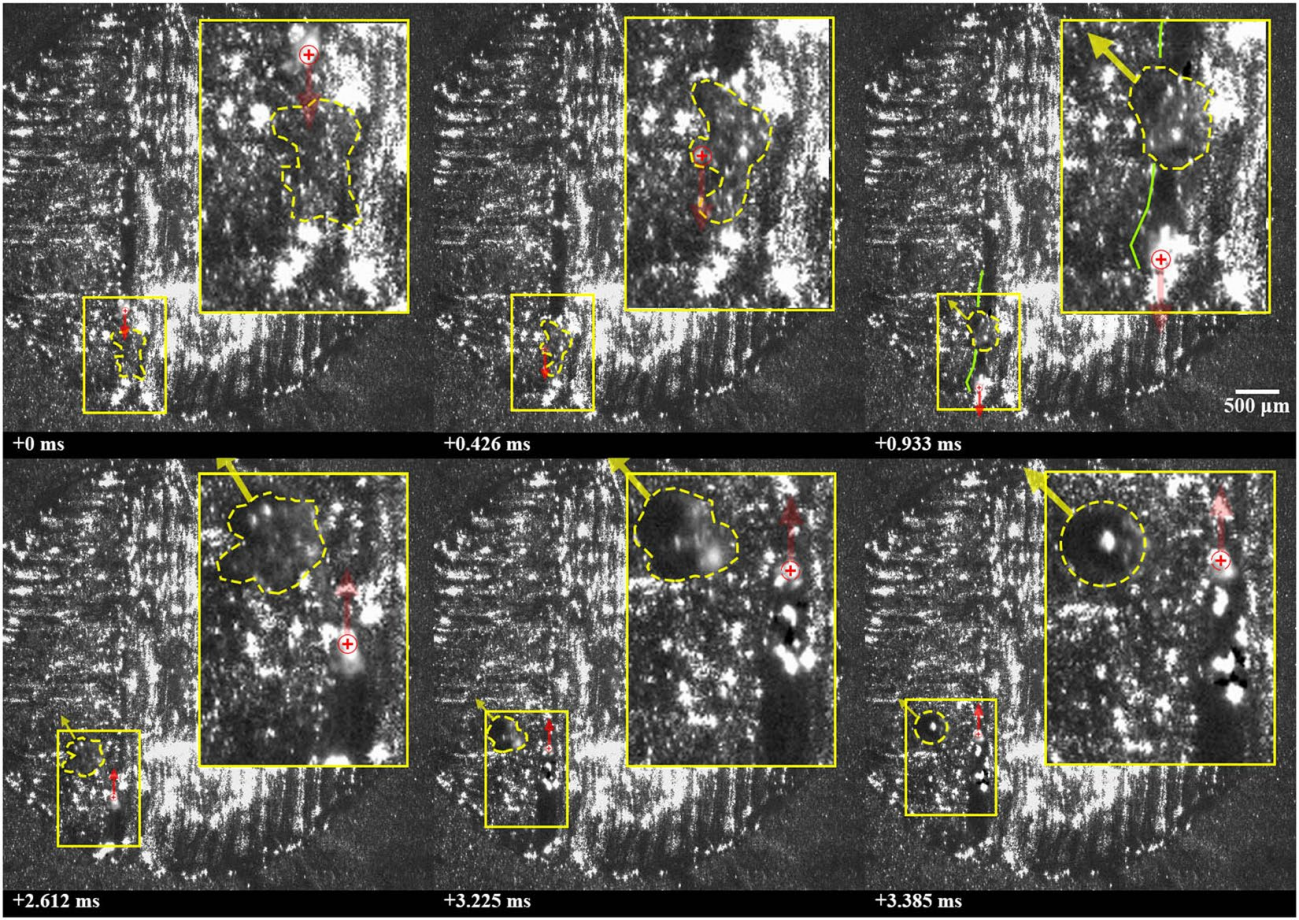
Interaction of a cluster of partially-sintered agglomerates (highlighted by yellow-dashed line) with the laser beam (red ⊕ symbol) followed by expulsion from the laser-interaction, and finally melting and coalescence of the cluster into a spheroidal particle. A solid, green line highlights the melt pool boundary in frame 3. Motion of the laser and particles are shown using arrows. A 2.5x magnified image is shown to the top right of each frame.

The plume also appears to contribute to heating, motion, and coalescence of partially-sintered agglomerates. This is most readily observed during laser glazing, where there is significantly less obstruction of the process by entrained powder. A laser glazing case is shown in Fig. 5 (See Supplementary Video S3), where a partially-sintered cluster of particles are initially resting atop the part surface and located directly in the path of a scanning beam. As the beam passes over the cluster, some melting and consolidation occurs and the cluster is ejected to the side of the melt. On the next hatch, the laser passes well to the right of the cluster with no obvious signs of direct laser beam interaction or interference. Note also that the position of the cylinder relative to the incoming laser beam would not result in specular laser reflections towards the cluster. However, rapid melting is observed—the cluster appears shiny and dark—and the shape becomes spheroidal.

**Figure 6:**
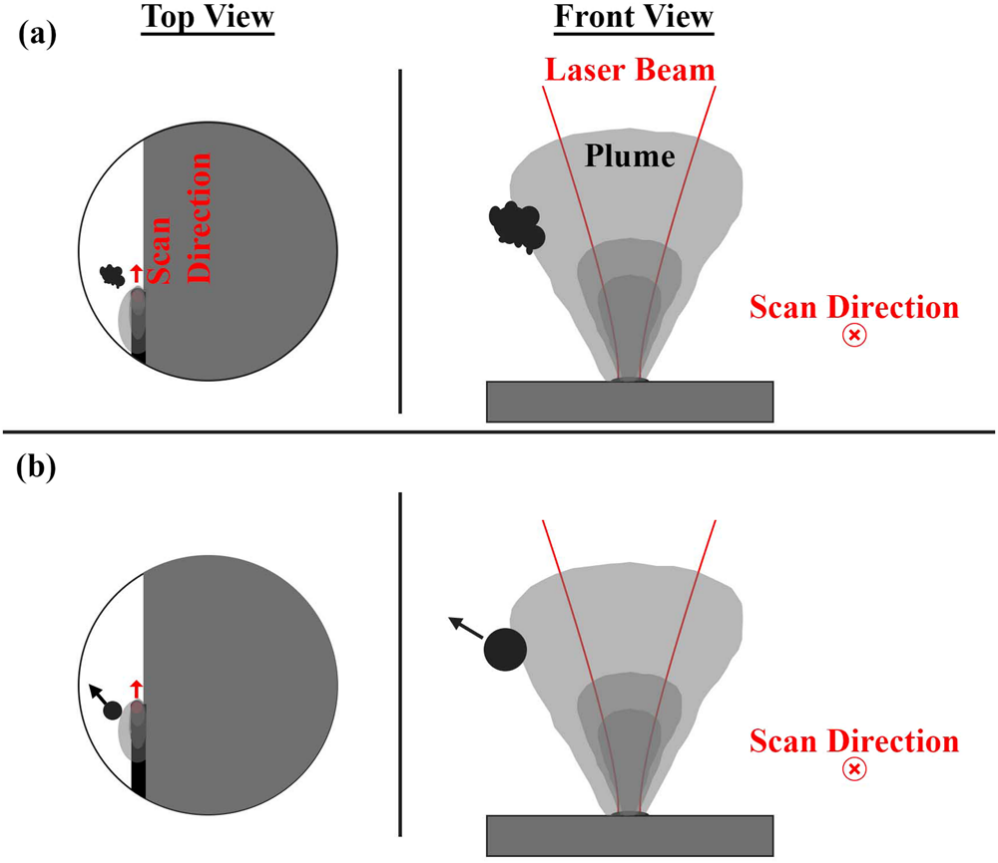
(**a**) A cluster of partially-sintered particles located in front of the laser and plume. (**b**) As the laser interaction zone passes near the cluster, a sufficiently hot, outer region of the plume heats the cluster causing it to coalesce into a spheroidal agglomerate. (Figure not drawn to scale).

It is proposed that the rapid melting and coalescence of the cluster observed in Fig. 5 is due to heating by the neighboring plume, which follows a similar argument by Bidare, *et al*.^7^ This mechanism is illustrated in Fig. 6. Given that the plume consists, at least in part, of vaporized constituents of the alloy, the gas temperature of the vapor must be greater or equal to the boiling point of the most volatile alloy constituent. In the case of nickel alloy 625, chromium represents the alloying element, having a concentration of more than a few percent, with the lowest boiling point (2,672 °C). This argument, together with the evidence provided in Fig. 5, thus provides a likely explanation for the formation of such large spheroidal agglomerates.

It is also noteworthy that just before the apparent interaction of the cluster with the vapor plume (between frames 3 and 4 of Fig. 5), the cluster moves at a speed of approximately 0.38 m/s. As the laser passes alongside the cluster (between frames 5 and 6 of Fig. 5), the now spherical and consolidated cluster rapidly accelerates to a speed of approximately 0.76 m/s with a slight change in trajectory. This again indicates that the acceleration of expelled particles is influenced by the vapor plume. It should also be noted that a component of the particle’s velocity appears oriented away from the surface of the cylinder; however, its magnitude cannot be measured with the available data.

### Interference of agglomerates with melt pool

**Figure 7:**
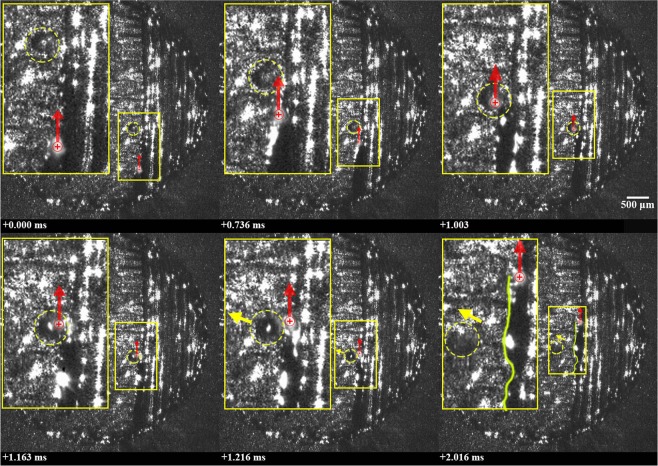
A large ejectum, which appears sintered to the underlying build, is expelled from the laser-interaction zone and appears to affect the melt pool geometry formed during processing. The location of the laser spot is shown using a red ⊕ symbol, the ejectum is highlighted using a yellow, dashed circle, and the melt pool geometry is highlighted in the last frame using a solid, yellow line.

The observation of large ejecta and the coalescence of the clusters during the PBFAM processes leads to an obvious question: what impact do such particles, once they land on the surface of build, have on subsequently-formed melt pools? It was observed that the majority of spatter particles do not affect melt pool geometry. However, in some cases, such to in frames 1–3 of Fig. 7 (See Supplementary Video S4), direct interference with the processing laser does occur. This figure, captured during laser glazing, shows a large, spherical ejectum, measuring approximately 150 μm in diameter, located in the path of a laser hatch. The ejectum appears to be sintered to the underlying part. As the laser beam passes over the ejecta, it melts and is ejected to the front, left of the melt pool. The interaction of the laser beam with the ejectum results in a loss of energy to the intended melt pool geometry leaving a perturbation in the track geometry.

**Figure 8:**
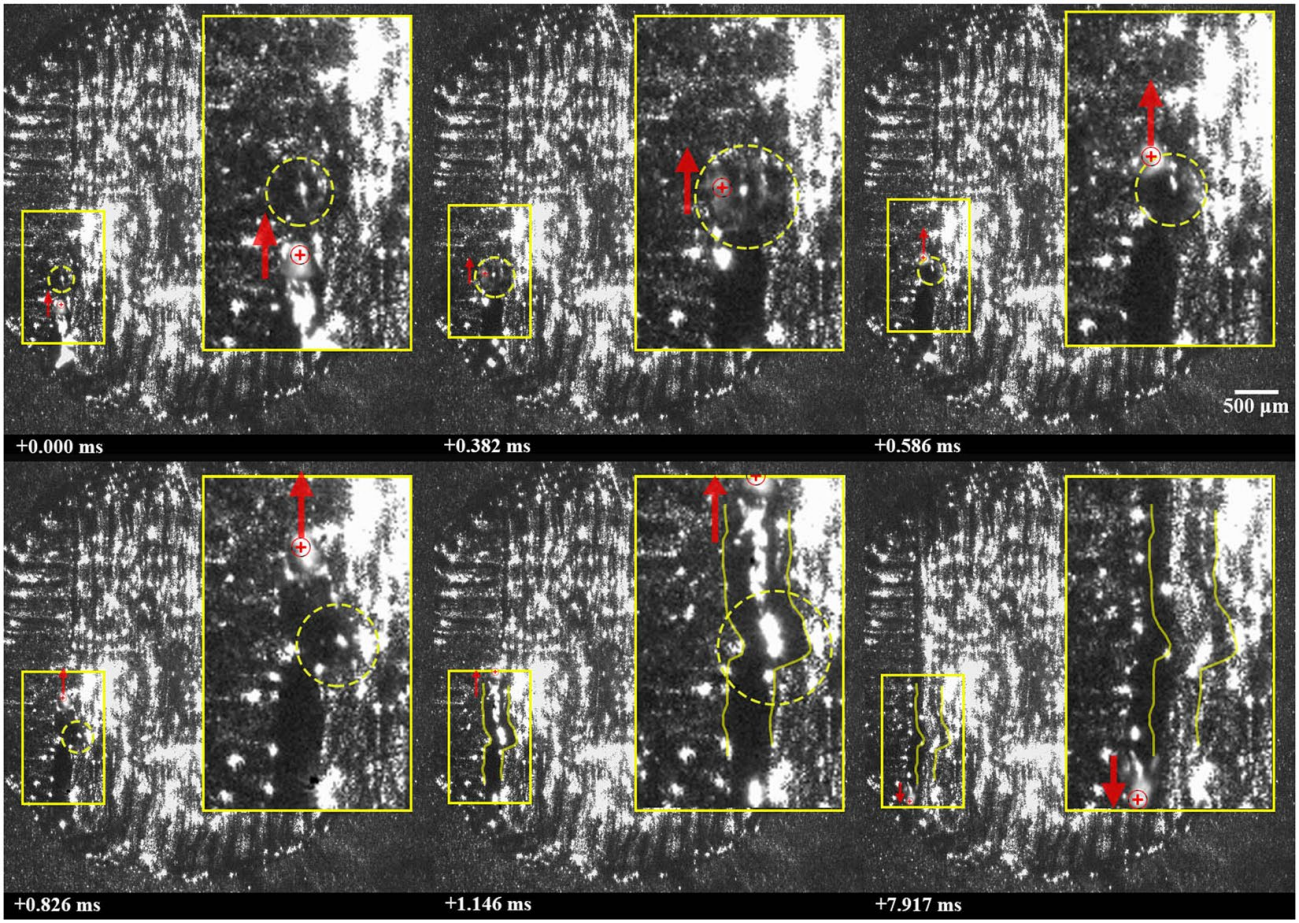
A large ejectum, which appears sintered to the underlying build, is incorporated into the melt pool and shifts its geometry. A 2.5x magnified image is shown to the top right of each frame. The location of the laser spot is shown using a red ⊕ symbol, the ejecta is highlighted using a yellow, dashed circle, and the melt pool geometry is highlighted in the last two frame using a solid, yellow line. Motion of the laser and particles are shown using arrows.

Another example of interference of ejecta with the geometry of a meltpool, observed during laser glazing, is provided in Fig. 8 (See Supplementary Video S5). Like the example illustrated in Fig. 7 (See Supplementary Video S4), a large, spherical ejectum is first located in the path of a laser scan; however, unlike the example in Fig. 7, this interaction leads to the incorporation of the ejectum into the melt track. As the laser beam passes over the ejectum (frames 2–3 of Fig. 8), it melts and is shifted by the laser interaction but is not expelled from the lase-interaction zone. Subsequently (frame 4–5 of Fig. 8), the ejectum becomes incorporated into the track geometry. However, due to the shift in position cause by the beam interaction and surface tension, the molten track is shifted to the right by approximately 120 μm. It is notable that this shift is on the order of the hatch offset distance. It is unclear if the particle is completely melted and incorporated into the melt.

The geometry of the neighboring laser hatch is shown in frame 6 of Fig. 8, where the prior, shifted track geometry is highlighted. It appears that the subsequent, neighboring track is affected by the prior track, as indicated by a slight shift in the right-most boundary of the subsequent track’s melt pool. We speculate that a variation in the degree of overlap caused by the described phenomena may result in a lack-of-fusion flaw.

**Figure 9:**
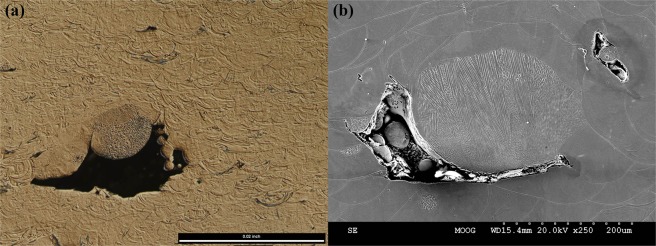
Examples of a flaw observed in PBFAM builds. (**a**) A partially-melted particle, on the order of 200 μm in diameter within a lack-of-fusion defect. (**b**) Microstructure suggestive of a partially-melted particles on the order of 250 μm in diameter neighboring a lack-of-fusion defect. Both (**a**,**b**) were observed in ASTM F75 CoCrMo alloy additively manufactured on an SLM280 system. The build direction is upward.

Ejecta, which are sintered to the substrate and in the path of a laser hatch, thus appear to be potentially harmful to the PBFAM process. On one hand, ejecta can be expelled from the laser-interaction zone after beam-interaction causing shadowing and energy loss to the melt track and a perturbation of the intended track geometry; and on the other hand, ejecta can be incorporated into the melt, increasing the intended mass of the melt pool and also perturbing the intended track geometry. In the first case, lack-of-fusion may occur due to the sudden drop in energy to the underlying powder/substrate. In the latter case, it is unclear if the ejecta is partially or fully melted. Incorporation of partially-melted ejecta into the melt may be one mechanism leading to the type of lack-of-fusion observed in Fig. 9, where large particle on the order of 200 μm (a) and 250 μm (b) in diameter neighbor irregular lack-of-fusion flaws in ASTM F75 cobalt-chromium-molybdenum alloy. Based on the dendritic microstructure of the particles and a chemistry (identified by energy-dispersive X-ray spectroscopy) consistent with the bulk material, it can be inferred that the particle was likely an ejectum which was incorporated into the melt pool during build up. Verifying this speculation is the subject of ongoing work.

## Conclusions

This work used direct, high-speed observations of a powder bed fusion process to classify and elucidate three mechanisms for the formation of large (much larger than the mass-median-diameter of feedstock powder) ejecta:

- collision and coalescence of ejecta from distant locations,
- collision and coalescence between neighboring, near-simultaneously-expelled ejecta, and
- coalescence of partially-sintered agglomerates by the vapor plume.

Evidence of interference of large ejecta with melt pool formation are also reported. Such interactions between large ejecta and the melt pool are shown to significantly perturb the intended track geometry in one of two ways. Ejecta can shadow or block the laser beam then be expelled from the laser-interaction zone, or can be incorporated into the melt pool and, under some circumstances, perturb the intended track geometry. It is argued that either mechanism may lead to lack of fusion and may explain the observation of stochastic (i.e. random or rogue) defects in PBFAM.

Additional work is required to further understand and model the mechanism which lead to the formation of large ejecta and the contribution of the identified mechanisms, together with melt ejection. The interaction of ejecta and the melt during scanning of hatches and contours also poses a critical and underexplored topic in powder bed fusion additive manufacturing. The physics underlying these interactions are not trivial and require significant additional development of current, multi-physics numerical models if the problem is to be addressed in practice. As such, no attempt has been made here to model these phenomena. Nevertheless, this wok provides insight into little-explored interaction mechanisms that should inform the development of physics-based simulations and the optimization of the PBFAM process.

Ongoing work seeks to quantify the likelihood of the detailed interactions and establish a causal link between interactions and flaw formation. While the captured and analyzed data includes over 70 gigabytes of video, this only represents 30 layers of buildup of a very small component (or roughly 6 meters of laser travel). Most PBFAM components are composed of many thousands of layers, each with a cross-section many times that of our test specimen, representing kilometers of laser travel. The captured subset is thus too small to extrapolate the likelihood of the identified mechanisms in commercial PBFAM processes. Nevertheless, each mechanism was identified at least once in a well-controlled environment during the course of our very small sampling period. So, while it appears that the identified mechanisms may be quite significant for commercial PBFAM processes, we are wary to extrapolate the likelihood of occurrence of the identified mechanisms for the formation of large ejecta or for the interference of large ejecta with melt pool formation.

## Methods

Experiments were carried on a 3D Systems ProX-320 PBFAM machine. The system utilized a 500 W (max power) fiber laser with an output wavelength of 1070 ± 10 nm. The feedstock material was nickel alloy 625 (Inconel® 625) powder with a particle size distribution of 11–53 µm. Powder was spread using a 3D Systems flexible, polymer recoater. A flow of argon gas traveled above and across the build plate, perpendicular to the recoating direction, at a speed of approximately 1.5 m/s, measured by a mini-vane anemometer. The build geometry consisted of a 5 mm diameter cylinder built using default processing parameters (Table 1). After recoating each 60 µm thick layer, a first contour was deposited using an offset distance of 68 µm from the geometric boundary of the cylinder, as defined in computer-aided design (CAD) software. This was followed by a second contour offset by 148 µm from the geometric boundary of the cylinder. Finally, the internal geometry of the part was built using a sequence of neighboring hatches, spaced 100 µm apart and alternating in direction. Between each layer, all hatches were rotated by 90 degrees.

**Table 1:** Processing parameters used for buildup of 5 mm cylinder in nickel alloy 625.

Parameter	Power (W)	Scan Speed (mm/s)	Hatch Spacing/Offset
Pre-contour 1	180	900	Offset 68 µm
Pre-contour 2	180	900	Offset 148 µm
Fill	235	900	Hatch 100 µm

**Figure 10:**
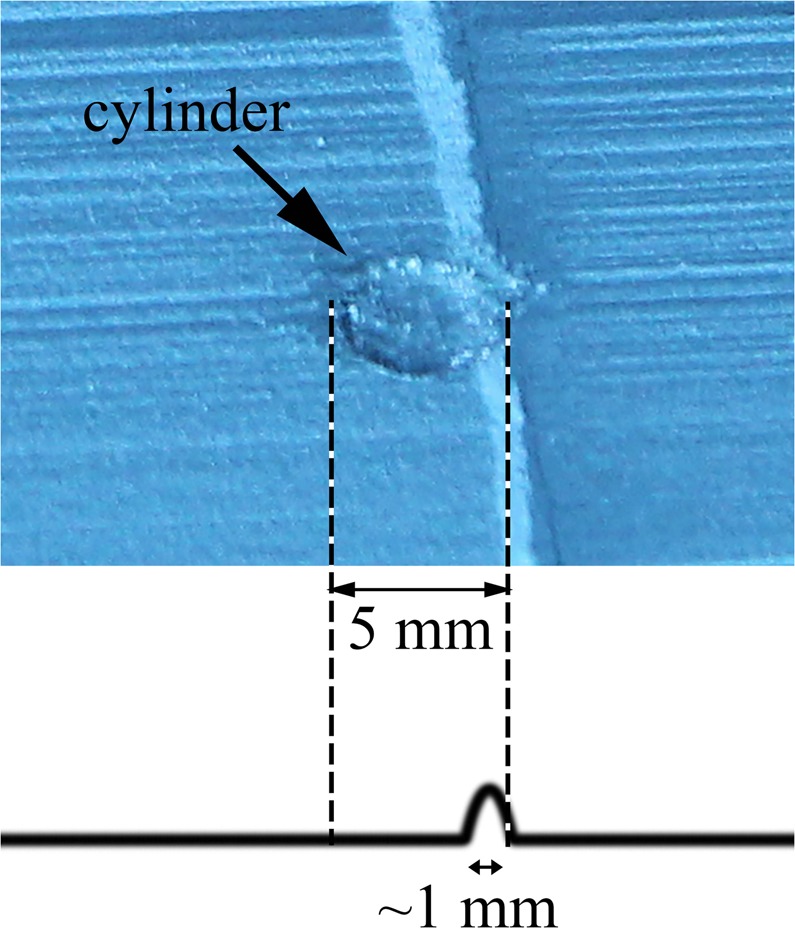
Image of the powder perturbation case together with an illustration of the approximate location and width of the cylinder and perturbation in the powder bed. Note recoating direction is from the left.

High-speed videos were captured during PBF using the processing parameters listed in Table 1, with two exceptions. In one case, denoted as the powder perturbation case, a powder perturbation was applied over the build by interrupting the recoating process over the location of the build cylinder (Fig. 10). Over the location of the cylinder an approximately 1 mm thicker powder layer was applied transverse to the recoating direction and with a width of approximately 1 mm along the recoating direction. In the second case, denoted as the glazing case, a layer was processed without powder recoating.

**Figure 11:**
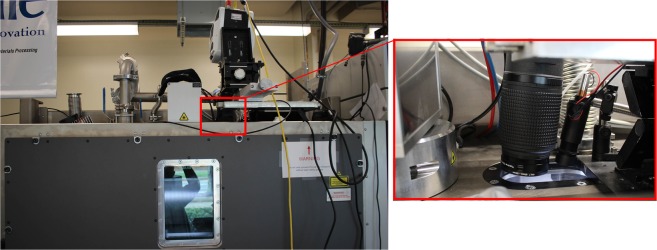
(left) Vision Research Inc. Phantom v1212 high speed camera mounted atop a ProX-320 system. (right) An illumination laser was directed through the same viewport and focused to a spot size of approximately 15 mm.

High-speed imaging was carried out through one of the two UV-fused-silica viewports atop the ProX-320 machine. As shown in Fig. 11, a Vision Research Inc. Phantom v1212 high speed camera and an illumination laser were positioned atop one of the two viewports of the ProX-320. Imaging optics included an optical train consisting of a Nikon 200 mm f/4 lens, (2) 2X teleconverters, and a single 1.4X teleconverter. The 200 mm lens f-stop was set to f/16. A cut-off filter was used to block wavelengths less than 375 nm and greater than 750 nm. An additional bandpass filter was used around 405 nm (10 nm full width at half maximum). Illumination of the powder bed was provided using a diode laser with a specified wavelength of 405 nm and a maximum output power of 900 mW. The illumination laser was focused by a series of optics to a circular spot approximately 15 mm in diameter and aligned to be approximately concentric with the build geometry. The 405 nm laser operated only during high-speed imaging for a time period of no more than 2 minutes before or after processing of each layer. An order of magnitude estimate for the temperature rise of the cylinder due to the 405 nm laser, based on input of ~5000 W/m^2^ over a 5 minute period into a 5 mm diameter nickel alloy 625 cylinder coupled to a 250 mm × 250 mm × 50 mm steel plate, results in a surface temperature rise less than 1 °C.

High-speed videos (512 × 512 resolution) were captured at 37,500 frames per second (27 µs per frame-spacing) with a frame exposure time of 25 µs. Within the high-speed video and images reported here, the argon gas across the build plate was oriented such that it traveled from the bottom to the top of each frame. The bottom of each frame represented the axis parallel to and closest to the front of the ProX-320 machine. Each video was manually post-triggered by observing the deposition process. For video captures of sequential layers, the build process was paused between layers to allow transmission of data from camera to external storage. Approximately 10 minutes were required to transmit 10,000 frames, with each layer requiring a minimum of ~11,000 frames. Over 70 gigabytes of video data were collected and manually analyzed—this represents buildup of approximately 30 layers.

## Supplementary information

Supplementary Video S1

Supplementary Video S2

Supplementary Video S3

Supplementary Video S4

Supplementary Video S5

## Data Availability

All data reported here has been approved for public release and is available.
